# Evaluation of Carotenoids Accumulation and Biosynthesis in Two Genotypes of Pomelo (*Citrus maxima*) during Early Fruit Development

**DOI:** 10.3390/molecules26165054

**Published:** 2021-08-20

**Authors:** Yihan Zhao, Xufeng Yang, Yuwei Hu, Qiuming Gu, Weiling Chen, Jiaqi Li, Xinbo Guo, Yutao Liu

**Affiliations:** 1 School of Food Science and Engineering, South China University of Technology, Guangzhou 510640, China; zhaoyihan19980515@163.com; 2 College of Food Science, South China Agricultural University, Guangzhou 510642, China; yangxufeng@stu.scau.edu.cn; 3 Key Laboratory of South China Modern Biological Seed Industry, Ministry of Agriculture and Rural Areas, National S&T Innovation Center for Modern Agricultural Industry, Guangzhou 510520, China; huyuwei0363@126.com; 4 Guangdong Lijinyou Agricultural Technology Co., Ltd., Meizhou 514743, China; guqm@163.com (Q.G.); chenweiling1011@163.com (W.C.); yr18031@163.com (J.L.)

**Keywords:** pomelo, carotenoids, biosynthesis, fruit development

## Abstract

Pomelo is rich in bioactive compounds (carotenoids, phenolics and essential oil) in the early stage of fruit development, but it is often wasted in the cultivation and management process. To gain an insight into the carotenoid metabolism pathway in pomelo, the carotenoid profiles and the expression patterns of carotenogenic genes were investigated in two genotypes of pomelo during early fruit development. The results showed that a higher carotenoid content was observed in honey pomelo as compared with golden pomelo, which may be related to different gene regulation mechanisms. Lutein, α-carotene, and β-carotene were the main carotenoids in pomelo young fruit, and lutein was the highest one. The accumulation of carotenoids during fruit early development in honey pomelo is related to the transcriptional regulation of *ZISO* and *LUT5*. In golden pomelo, the rate-limiting gene for carotenoids is *PDS* and *ZDS*. In addition, the expression of seven genes except *CRTISO* in honey pomelo was higher than that in golden pomelo. The results are helpful to further clarify the regulatory mechanism of carotenoid accumulation during early fruit development and provide a direction for the high-value utilization of young fruits in pomelo.

## 1. Introduction

Pomelo belongs to the genus Citrus, and it is widely cultivated in the world [1]. China is an important diversity center of the genus Citrus, especially in pomelo germplasm [2]. Pomelo is one of the best well-known fruits because of its nutritional and medicinal value [3], which is rich in phytochemicals (including carotenoids, phenolics and essential oil) [2,4]. Honey pomelo (*Citrus grandis* cv. miyou) (HP) and golden pomelo (*Citrus maxima* (Burm.) Merr. cv. Meizhou Yu) (GP) are the two most widely planted genotypes and the two most predominantly traded species in Southern China [5,6]. According to the data released by the Food and Agriculture Organization of the United Nations, the planting area of pomelo crops in China has reached 93 thousand hectares, with an annual output of 5.08 million tons in 2019, accounting for more than half of the world's output of pomelo crops, and the output is still increasing year by year. In order to obtain better quality pomelo fruit, reasonable thinning of pomelo fruit should be carried out during fruit early developmental stages. For young fruit that is removed, they are usually discarded. However, these young fruits also have great value for exploitation. At present, the research on pomelo mainly focuses on pomelo (*Citrus paradisi* Macf.) [4], and there are few studies on the Chinese indigenous pomelo crops. Most studies focus on the ripening fruit of pomelo [1], but there are still few studies on the growth process of pomelo.

Carotenoids are important natural pigments in most plants and some shellfish animals [7]. Natural carotenoids provide yellow to red colors for fruits and vegetables, which are rich in dark green fruits and vegetables [8]. Carotenoids are isoprenoid compounds, mainly divided into two groups: carotene and lutein [9], which are synthesized in various types of plant plastids [10]. In the straight-chain carotenoid synthesis pathway, two Geranylgeranyl diphosphate (GGPP) molecules are synthesized into 15-cis-octahydro-lycopene by the action of octahydro-lycopene synthase (PSY) [11]. Octahydro-lycopene undergoes the combined action of octahydro lycopene dehydrogenase (PDS), ζ-carotene isomerase (Z-ISO), ζ-carotene dehydrogenase (ZDS), and carotenoid isomerase (CRTISO) to produce pink all-trans lycopene [8]. In the presence of lycopene β-cyclase (LCYB) and lycopene ε-cyclase (LCYE), carotenoids are epoxidized and the anabolic pathway is branched to synthesize α-carotenoids and β-carotenoids, respectively [8]. On this basis, a series of carotenoids are produced [12]. Some kinds of carotenoids, such as α-carotene and β-carotene, can be converted into vitamin A in the human body, which is very important to human health. Carotenoids can also be used as biochemical synthetic precursors of antioxidants [13], which play an important role in reducing chronic diseases [14]. Products rich in carotenoids have been shown to play a beneficial role in reducing cancer and cardiovascular and cerebrovascular diseases [15]. As an important nutritional supplement, carotenoids are obtained mainly by eating fruits and vegetables in daily life [15]. Citrus fruits are rich in carotenoids, which is one of the main sources of carotenoids in the human diet [4,16]. Past studies have shown that pomelo fruits such as honey pomelo are rich in carotenoids and have high development potential [17]. 

Pomelo by-products, as represented by the peel, have been shown to have high nutritional value and have a wide range of applications in food processing, chemicals, and pharmaceuticals, enabling the recovery or production of valuable products [18]. It can be directly used to produce products such as peel preserves, teas, jams, etc. [19]. It has also been used to manufacture adsorbents, bioethanol, etc. [20,21]. Pomelo peel extracts and isolated pure compounds also have a wide range of biological activities [22,23,24,25]. The epidermis of young pomelo fruit is dark green, the juice vesicle is not yet developed. Most of the carotenoids are found mainly in the epidermis of young pomelo fruit [8]. Previous studies have shown that the peel of pomelo is rich in carotenoids [4]. As a by-product of the pomelo production process, the young pomelo fruit is also often neglected and has similar biological structural properties to the peel before the pulp has developed, which will cause pollution and waste if not handled properly. Young pomelo fruits have a large specific surface area, which can infer that their carotenoid content is high and has a high utilization value. In order to investigate the changes of carotenoid accumulation during pomelo fruit development and reveal the relationship between carotenoid accumulation and related gene expression during pomelo fruit development, two popular genotypes of young pomelo fruits with different developmental stages were compared in this study. 

## 2. Results

### 2.1. Changes of Carotenoid Profiles in Pomelo during Early Fruit Development

**Figure 1:**
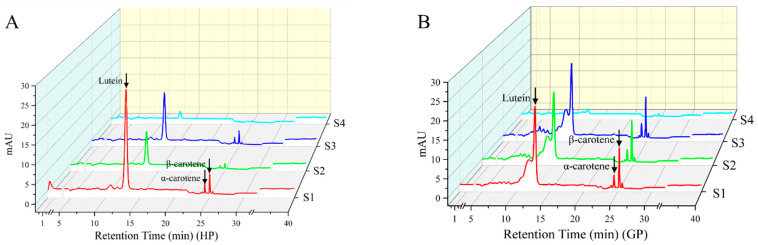
HPLC profiles of carotenoid compositions in young pomelo fruits. (**A**) Honey pomelo; (**B**) Golden pomelo. S1~S4 indicate different developmental stages.

The carotenoids in pomelo were determined by high-performance liquid chromatography (HPLC); ten common carotenoid monomer components were used as the standards for qualitative and quantitative analysis. Three carotenoids (lutein, α-carotene, and β-carotene) were both detected in two genotypes of pomelo, and the changes of chromatography profiles during fruit development as shown in Figure 1.

**Figure 2:**
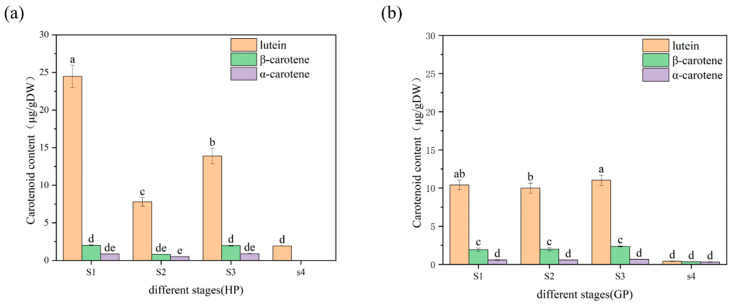
Changes in carotenoids content and compositions in pomelo young fruit. (**a**) Changes in carotenoids content and composition of honey pomelo during four developmental stages; (**b**) Changes in carotenoids content and composition of golden pomelo during four developmental stages. Different letters indicate significant differences between values (*p* < 0.05).

As shown in Figure 2, the content of total carotenoids in honey pomelo was the highest at the S1 stage (27.36 ± 1.46 μg/g DW). The content of carotenoids sharply declined to the lowest at the S2 stage (9.11 ± 0.58 μg/g DW), which was 33% of the initial contents. Then, the carotenoids content was recovered to 61% of the initial at the S3 stage and decreased to the lowest at the S4 stage (1.93 ± 0.28 μg/g DW), which was 7% of the initial content in honey pomelo. However, the total carotenoids content of golden pomelo showed no significant difference in the first two stages but increased in the third stage and decreased rapidly in the last stage. The carotenoids content was lowest at the S4 stage (1.07 ± 0.03 μg/g DW) and highest at the S3 stage (14.03 ± 0.61 μg/g DW) in golden pomelo. During the whole early development stages, the average carotenoids in honey pomelo and gold pomelo were 13.78 ± 10.88 μg/g DW and 10.20 ± 6.11 μg/g DW, respectively. In general, the carotenoids content of honey pomelo was higher than that of golden pomelo. During the four developmental stages of honey pomelo, the total carotenoids content showed an overall decreasing trend, and a similar pattern was observed in golden pomelo (Figure 2). However, the change in the content of golden pomelo tended to be stable. In the first three periods, the mean value of total carotenoids content was slightly higher than the initial period with fruit development. In S1 and S3 stages, the total carotenoids content of honey pomelo was significantly higher than that of golden pomelo.

Lutein was the highest carotenoid compound in the two genotypes and followed by β-carotene, which presented similar trends as total carotenoids in pomelo development of the two genotypes. In honey pomelo, lutein contributed more than 80% (arranged from 82.89% to 100%) to total carotenoids, the highest content was 24.48 ± 1.47 μg/g DW at the S1 stage. β-carotene also contributed 7.37% to 11.72% for total carotenoids in the first three periods of honey pomelo, whereas α-carotene contributed the lowest for total carotenoids and ranged from 3.14% to 5.56%. In golden pomelo, lutein contributed 38.70% to 80.70% for total carotenoid, and the highest content was 11.02 ± 0.68 μg/g DW at S3 stage, which was lower than 50% of S1 and similar with S3 in honey pomelo. β-carotene contributed more than 15% to total carotenoids in golden pomelo, and the highest content was 2.35 ± 0.06 μg/g DW at the S3 stage, which was 117% of the highest content in honey pomelo. The content of α-carotene was also the lowest in golden pomelo, which contributed about 4% for total carotenoids and had no significant change in the first three periods, but its contribution increased to 29.83% in the S4 period due to a significant decrease in the content of other components. The results showed that two genotypes of pomelo had different changing patterns of carotenoid profiles and contents during early fruit development. During the first three periods, the highest carotenoids content was found in honey pomelo, but golden pomelo changed more stable. 

### 2.2. Differential Genes Expression in Carotenoid Biosynthesis Pathway

**Figure 3:**
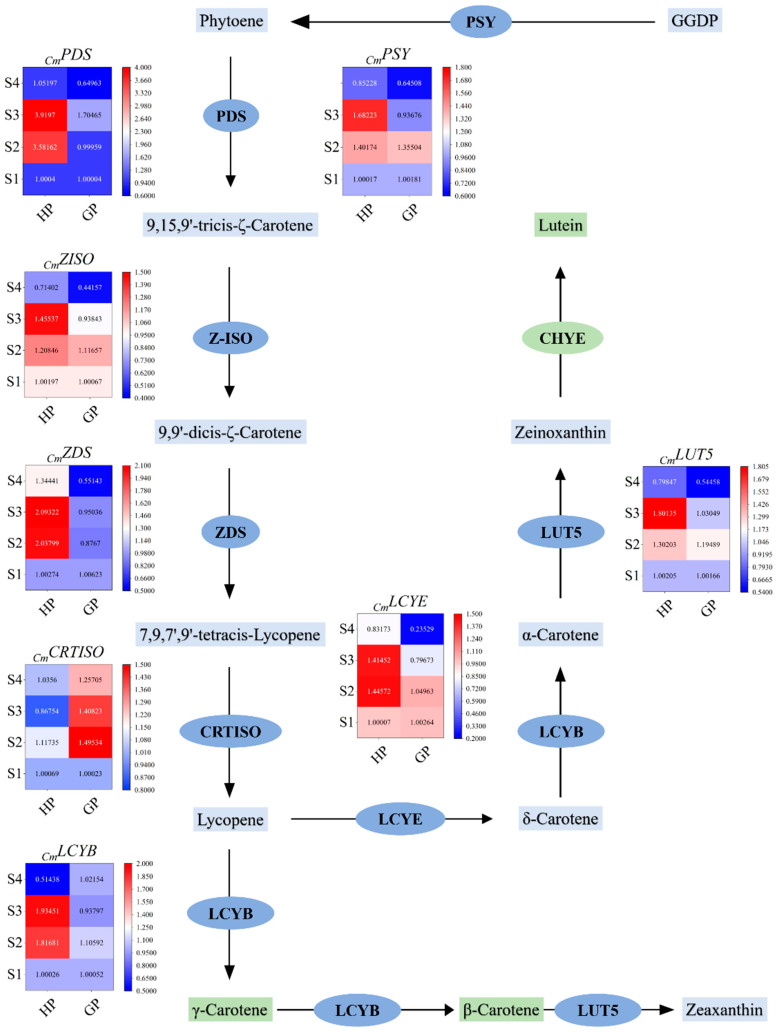
Carotenoid biosynthesis pathways and related gene expression levels during early fruit development. Values are showed as the means ± SD (n = 3). GGDP, geranylgeranyl diphosphate; *PSY*, phytoene synthetase; *PDS*, phytoene desaturase; *Z-ISO*, ζ-carotene isomerase; *ZDS*, ζ-carotene desaturase; *CRTISO*, carotenoid isomerase; *LCYE*, lycopene ε-cyclase; *LUT5*, β-ring hydroxylase; *LCYB*, lycopene β-cyclase.

In order to understand the accumulation mechanism of carotenoids in pomelo development, some key genes were selected from the carotenoid biosynthesis pathway for further study. The rough biosynthetic pathway of carotenoids and the varied relative expression levels of related genes are shown in Figure 3. The expression of genes involved in the carotenoid biosynthesis pathway at four development stages of pomelos was determined by RT-qPCR. In general, the relative expression levels of genes varied during fruit development (Figure 3). The expressions of 8 genes involved in the carotenoid biosynthesis pathway were analyzed in the young fruit of golden pomelo and honey pomelo at four different developmental stages. As shown in Figure 3, the expression of *PSY* in the two genotypes was similar in the first two periods, both of which were up-regulated in the S2 period. However, the expression level of honey pomelo continued to increase in the S3 period, while the expression level of gold pomelo was on the contrary, and the gene expression trend of *LUT5* also showed a similar trend. 

The expression trend of *PDS* in the two genotypes of pomelo was different and the expression of honey pomelo was consistently higher than that of golden pomelo. The expression of *PDS* in honey pomelo was very low at the S1 stage but increased significantly at S2 and S3 stages compared with the initial level. The expression level of golden pomelo was very low in the first two periods and increased in the S3 period, finally the same was significantly decreased in both genotypes in the S4 period. The expression pattern of *ZISO* in both genotypes was similar in the first two periods, it increased rapidly in the S2 period. However, during the S3 period, honey pomelo continued to be up-regulated while golden pomelo started to be down-regulated, but both showing a down-regulation trend in the S4 period. The expression of *ZDS* in golden pomelo was relatively low and showed an opposite trend to honey pomelo in the S2 period (Figure 3), but showed an up-regulation followed by a down-regulation pattern in the last two periods. The expression of *LCYB* was highly expressed in honey pomelo in the S2 period, then continuously up-regulated and rapidly in the S4 period. while fluctuating in a smaller magnitude in golden pomelo. In the first three periods, *LCYE* and *CRTISO* expression trends were similar in both genotypes, with significant up-regulation in the S2 period and followed by rapid down-regulation, the difference is that *CRTISO* expression was higher in golden pomelo than in honey pomelo. In addition, in the S4 period, *LCYE* continued to be down-regulated in both cultivars, while *CRTISO* was down-regulated in golden pomelo and showed an up-regulation trend in honey pomelo.

### 2.3. Correlations among Compositions and Gene Expressions 

**Figure 4:**
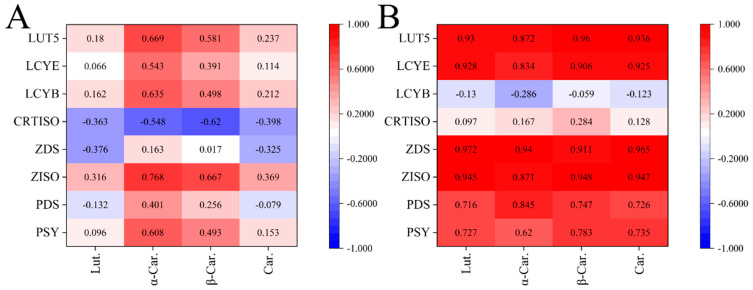
Pearson correlations between carotenoids (**A**,**B**) composition contents and their gene expression levels. (**A**) The correlations of honey pomelo. (**B**) The correlations of golden pomelo. “Lut.” stands for lutein; “α-Car.” stands for α-carotene; “β-Car.” stands for β-carotene; “Car.” stands for total carotenoids.

The correlation analysis of the measured components and relative expression levels of key genes was performed using Pearson’s multiple comparison post-test. Figure 4 was made by Origin 2018. During the young fruit development, the relative expression of most genes was correlated with carotenoid components. In honey pomelo, the relative expression of most genes was not highly correlated with carotenoid components. In golden pomelo, most genes were highly positively correlated with carotenoid composition (r > 0.9). Expression of *ZDS* was significantly positively correlated with the content of all carotenoid components in golden pomelo (r > 0.9), while *LUT5*, *LCYE*, and *ZISO* were correlated with the content of all carotenoid components (r > 0.8), and *PDS* and *PSY* were correlated with the content of all carotenoid components with correlation coefficients above 0.7 and 0.6, respectively. The expression of the *LCYB* gene was negatively correlated with all carotenoid components, and the positive correlation between *CRTISO* and the components was also low. In honey pomelo, *ZISO* showed the highest correlation with α-carotene and β-carotenoid contents (r = 0.768, 0.667).

## 3. Discussion

### 3.1. Variations in Carotenoid Accumulation and Gene Expression

Regulation of carotenoids in plants is a complex mechanism due to high variation between different genotypes, developmental stages, and plant tissue [4,26]. It was found that two genotypes of pomelo young fruit displayed different expression patterns of carotenoid genes during early development as shown in Figure 4. The expression levels of most carotenoid genes except *CRTISO* in honey pomelo were higher than those in golden pomelo during early development. In honey pomelo, transcript levels of these genes except *CRTISO* displayed an uptrend during early development, especially *PDS*, which had higher expression levels than other genes. On the other hand, the expression levels of these genes, except *PDS* and *ZDS*, displayed an uptrend first and then declining during early development in golden pomelo. During plant fruit development, the regulation of carotenoid genes is a mechanism among various factors affecting carotenoid accumulation [12]. The expression levels of carotenoid biosynthetic genes except for *CRTISO* in honey pomelo were higher than those in golden pomelo during early development, which may be one of the reasons for the higher total carotenoids content in honey pomelo than that in golden pomelo. This result showed that the expression of carotenoid genes played a vital role in the accumulation of carotenoids, which has also been proved in the development of peach [27], mango [28], *Cucurbita pepo* [29], and bilberry [30].

During the S2 stage of honey pomelo, all the eight genes showed up-regulation to different degrees, especially the *PDS* up-regulation by 3.5 times, while the *CRTISO* up-regulation was not obvious. In the S3 stage, except for *CRTISO* and *LYCE*, the other genes continued to increase, and in the S4 stage, the relative expressions of all genes except CRTISO showed a decreasing trend, while the carotenoids content showed a trend of first decreasing, then increasing and finally decreasing to the lowest point. This indicated that the variation in carotenoids content and composition in young pomelo fruit could not always be described by the transcriptional regulation. Similar situations also occurred in sweetcorn [31], potato [32], and watermelon [33]; the changes of carotenoids content did not completely follow the expression pattern of most genes. It is speculated that there is feedback inhibition in the carotenoid biosynthesis pathway of pomelo or carotenoids that were catabolized into other pathways during the S2 stage [28]. The increased activity of some genes promoted the supply of ABA biosynthesis precursors, resulting in a decrease in carotenoids content. During the S3 stage, due to the continuous up-regulation of related genes and relatively low carotenoids content, carotenoids in young fruit of honey pomelo began to accumulate again, while at the S4 stage resulted in a decrease in carotenoids content due to the down-regulation of most genes. In golden pomelo, the carotenoid content did not change significantly in the first two periods, increased in S3 and decreased to a minimum in S4, which was consistent with the changing trend of *PDS* and *ZDS*, indicating that *PDS* and *ZDS* may be the rate-limiting gene and the expression of the upstream gene could effectively regulate the accumulation of carotenoids in golden pomelo during fruit early development [27].

### 3.2. Alterations of Carotenoid Profiles and Contents during Fruit Development

Citrus fruits are rich in carotenoids, which are not only high in content but also have many kinds [34], so it has always been a good material for studying the physiology and regulation of carotenoid metabolism in plants [4]. Pomelo contains two kinds of natural pigments with different properties, one is the fat-soluble carotenoid, the other is the water-soluble yellow pigment [34]. Carotenoid is one of the main pigments in pomelo fruit, its content and composition are not only related to the color of pomelo appearance and edible nutritional value [35] but also related to the color of pomelo processing products, which is an important factor to determine the color and biological activity of pomelo fruit. The significant variation in pomelo flesh color is attributed to differences in carotenoid composition and content. Up to 11 different carotenoids have been identified in Yuhuan and Chuzhou Early Red pomelos [4]. It was found that the red pulp color of Tubtim-Siam pomelo was related to the proportion of the main pigments which include, lycopene, β-carotene, lutein, and zeaxanthin [35]. This study also confirmed that there were carotenoids in pomelo young fruit. During the early fruit development, the color of the two pomelo genotypes did not change significantly, the corresponding carotenoid species and main component also did not change. The total carotenoids content of the two genotypes was the highest at 27.36 μg/g DW and 14.03 μg/g DW, which might help people reach the recommended daily intake of vitamin A (900 micrograms of retinol equivalents/day) by eating a product made from the extract of pomelo young fruit [36]. 

The species of carotenoids detected in the two genotypes were consistent: the main component was lutein. Xu et al. [4] found that lutein accounted for the majority of colored carotenoids in ordinary citrus tissues, while lycopene was not observed and only low levels of α-carotene and β-carotene was observed, which was consistent with our results. However, β-carotene is the main ingredient in yellow/orange-colored fruits such as mango [28] and loquat [37], presumably due to species differences. Carotenoids are naturally found in the green tissues of all plants, and they play essential roles in the development, photosynthesis, and production of phytohormones such as abscisic acid [29]. The biosynthesis of carotenoids is regulated throughout the life cycle of plants. The dynamic changes of carotenoid components during fruit development match the general developmental requirements and respond to environmental stimuli [7]. A large number of young fruits fall off naturally during citrus cultivation and are usually disposed of as waste. The effective use of the active components of pomelo fruit can not only reduce the environmental pollution caused by the accumulation of young fruit but can also make full use of the abandoned natural resources and can greatly improve the economic added value of pomelo, with good economic and social benefits. In this study, it was found that pomelo fruit was rich in carotenoids, among which lutein was the main component, up to 24.48 μg/g DW. 

Thus, in view of the above, it can be said that pomelo young fruit is a potentially valuable source of industrially important carotenoids. According to the results of this study, it was found that the variation trend of carotenoids content in young fruits of honey pomelo and golden pomelo was similar in four developmental stages, but the range of variation was larger in honey pomelo and decreased significantly in the second stage. Moreover, the carotenoids content of both genotypes reached the minimum value in the last period. Therefore, the first or third stage of pomelo should be selected as the material for carotenoid extraction. However, since the content of carotenoids in golden pomelo varies little, the three stages can be used as extraction materials. Therefore, it is of great theoretical and practical significance to study the changes of carotenoids in pomelo fruit during development, both from the perspective of comprehensive utilization of pomelo resources to extract active ingredients and from the perspective of exploitation and utilization of pigment resources, which can provide the theoretical basis for scientific exploitation and utilization of pomelo fruit.

## 4. Materials and Methods

### 4.1. Plant Materials and Sample Collection

**Figure 5:**
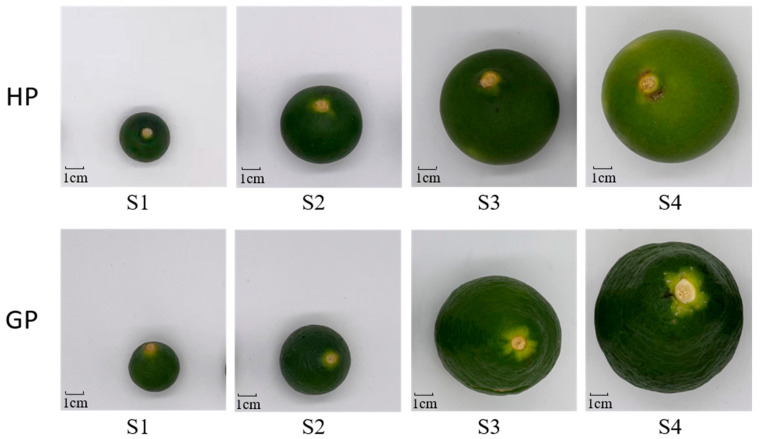
Pomelo fruit development stages. S1–S4 four sampled stages during fruit development. The upper fruits are “Golden pomelo” (GP) and the down fruits are “Honey pomelo” (HP).

Two genotypes of young fruits (Honey Pomelo and Golden Pomelo) were cultivated and collected from the Meizhou Honey Pomelo National Industrial Park (near latitude and longitude 24.270917° N; 116.08771° E; Meizhou, China). The fruit trees are planted in an orchard managed by a smart agriculture monitoring system, which monitors and adjusts the soil conditions in real time, well ensuring the controllability and stability of the samples. Young pomelo fruits were collected from late April to early May 2021, and young fruits with uniform color and fullness were selected as samples. The average monthly temperature was 28 °C during the early fruit developmental period. Fruits of each variety were sampled evenly from three 10-year-old pomelo trees with the same growth environment and maintaining the same genotype. Young pomelo fruits developed to 2 cm, 5 cm, 8 cm, and 10 cm in diameter were divided into four periods S1, S2, S3, and S4, respectively (Figure 5), and more than 30 fruits were collected at each developmental stage. Fresh young fruits were all washed, cut into thin slices of 2–3 mm, frozen in liquid nitrogen immoderately, and more than 50 g of each sample was taken evenly from the mixture for both genotypes and stored at −80 °C for RNA extraction and gene expression analysis. The remaining samples were dehydrated and dried in a vacuum freeze dryer. After dehydration and drying, the samples were crushed with a liquid nitrogen grinder and stored in a −20 °C refrigerator for carotenoids extract and analysis. 

### 4.2. Extraction of Carotenoids

The extract method of carotenoids in young pomelo fruits was followed by previously reported in our lab [38]. Briefly, pomelo fruit powder was mixed with 95% ethanol, 0.3 M NaCl, 0.5 M pyrogallol, 1 M ascorbic acid, and 10.7 M KOH, and then the mixture was saponified at 75 °C for 45 min. After saponification, the mixture was extracted with n-hexane/ethyl acetate (9:1 *v*/*v*) six times, and the organic supernatants were merged and dried with nitrogen gas. The residues were dissolved in methyl tert-butyl ether (MTBE) with 1% 2,6-Di-tert-butyl-4-methylphenol (BHT) for carotenoids analysis. All the samples were three replicates for carotenoids extraction.

### 4.3. Carotenoid Analysis

The analysis of carotenoids refers to the methods previously reported in our lab [38]. Waters high-performance liquid chromatography (HPLC) system (Waters Corporation, Milford, MA, USA), a YMC carotenoid 30 column (5 μm, 4.5 × 250 mm) with 25 °C temperature and a photodiode array detector were applied in the measurements. The mobile phase A was 0.1% (*w*/*v*) BHT and 0.05 M ammonium acetate in 97% (*w*/*v*) methanol–water, and the mobile phase B was 0.1% (*w*/*v*) BHT in methyl tert-butyl ether. The gradient elution sequence was as follows: 0 to 18 min, 0−20% B; from 18 to 20 min, 20−50% B; from 20 to 25 min, 50–90% B; from 29 to 29.5 min, 90−10% B; from 29.5 to 40 min, 10−0% B. The flow rate was one milliliter per minute. The UV absorbance was set at 450 nm. Carotenoid standards were purchased from CaroteNature (CaroteNature, Münsingen, Switzerland). Qualitative and quantitative analysis of carotenoids was carried out by comparing the retention time and peak area with the standards. Data were expressed as micrograms per gram in the dry weight of the sample and showed as µg/g DW (means ± SD).

### 4.4. RT-qPCR Assay

Total RNA of young pomelo fruits was isolated using the Plant RNA Kit (TIANGEN Biotech Co. Ltd., Beijing, China) and reversed to cDNA by the FastKing RT Kit with gDNase (TIANGEN Biotech Co. Ltd., Beijing, China), respectively. Quantitative real-time PCR was operated with the SYBP^®^ Premix Ex TaqTM Kit (TIANGEN Biotech Co. Ltd., Beijing, China) by the LightCycler^®^ 480 Real-Time PCR System (F.Hoffmann-La Roche Ltd., Basel, Switzerland). *ACT* (GenBank ID: 18038212) was selected as a reference gene and the primer sequences of genes in this study are listed in Supplementary Materials (Table S1). Ct values were processed with the 2^−ΔΔCt^ method for calculating relative expression. Results were performed as the means ± standard deviation (SD).

### 4.5. Statistical Analysis

The data were analyzed using the SPSS 26.0 software (IBM, Endicott, NY, USA). A one-way ANOVA followed by Duncan’s multiple comparison post-test was used to compare differences among data of three biological replicates from different mature stages. The correlation analysis was performed using Pearson’s correlation by SPSS 26.0. A *p*-value less than 0.05 was considered significant. All measurements were performed in triplicates and results were expressed as the means ± SD (n = 3).

## 5. Conclusions

In summary, during the early development of pomelo carotenoid gene expression plays a crucial role in carotenoids accumulation. The regulation of gene expression at the transcriptional level is the key regulatory mechanism for carotenoids accumulation in pomelo. The higher expression levels of key genes in the carotenoid biosynthesis pathway would cause the higher total carotenoids accumulation in young pomelo fruits. Lutein, α-carotene, and β-carotene are the main carotenoids in pomelo fruit. Young pomelo fruits would be an important stage for carotenoids accumulation during fruit development. The current exploitation of young pomelo fruit is relatively small, and if it can be used to develop fortified or functional foods and nutritional foods it will further increase the added value of pomelo and will benefit the development of the food industry. The present results established a theoretical data basis for the cultivation and management of pomelo and the utilization of pomelo young fruits.

## Data Availability

The data presented in this study are available in article.

## Supplementary Materials

The following are available online, Table S1: Primer sequences of genes for Real-Time PCR.

## Sample Availability

Not available.
