# Supplementary material for: Evaluation of Carotenoids Accumulation and Biosynthesis in Two Genotypes of Pomelo (Citrus maxima) during Early Fruit Development
Source: Molecules. 2021 Aug 20;26(16):5054. doi: 10.3390/molecules26165054 (PMC8400066; doi:10.3390/molecules26165054)
Supplement: Supplementary file 1 [file molecules-26-05054-s001.zip › molecules-1347874-supplementary.pdf]

Table S1. Primer sequence of related genes for Real-Time PCR

Gene Name	Gene ID	Prime Direction	Primer Sequence 5'-3'
CRTISO	LOC18043114	Forward	TCCAAAGTCCTACCGAGAT
		Reverse	GGTCCCAGTCATTCACAAC
GGPPS	LOC18042186	Forward	TTCCACTTCCACATCCTGCTA
		Reverse	TCGCCACTGACTGACTTCCT
LCYB	LOC18031713	Forward	GGTTCTTGATGCTACGGGATT
		Reverse	CAAACGGGTGCTCTTCTAC
LCYE	LOC18056072	Forward	AAAGCATTACGGAATCTAC
		Reverse	GGACAGAAACTTTGGGAC
LUT5	LOC18039008	Forward	GGAGCTGCAGTTTCATGAGG
		Reverse	ACTGCAGCTGATGTTTCGTG
PDS	LOC18034521	Forward	CTGGATTACAACCCGGCATG
		Reverse	AATCTTCTGTTGACGGTGCG
PSY	LOC18039146	Forward	TGGAGAAGTGGCCATGTCTT
		Reverse	CGCAAACTTCTCCACAACGA
ZDS	LOC112098231	Forward	ACAGAGAAGGGCAAGGTTCA
		Reverse	AGGTCCCTCACGGTACAAAG
ZISO	LOC18046078	Forward	GGGAAACTGGGGTCATGAGA
		Reverse	GTTCGCCTCTTCACAGCTTC
ACTIN	LOC18038212	Forward	GCTATCCAGGCTGTGCTTTC
		Reverse	AACAATTTCCCGCTCAGCAG
